# Supplementary material for: Human Milk from Previously COVID-19-Infected Mothers: The Effect of Pasteurization on Specific Antibodies and Neutralization Capacity
Source: Nutrients. 2021 May 13;13(5):1645. doi: 10.3390/nu13051645 (PMC8152997; doi:10.3390/nu13051645)
Supplement: Supplementary file 1 [file nutrients-13-01645-s001.zip › nutrients-1193663-Supplement file.pdf]

Supplementary Materials

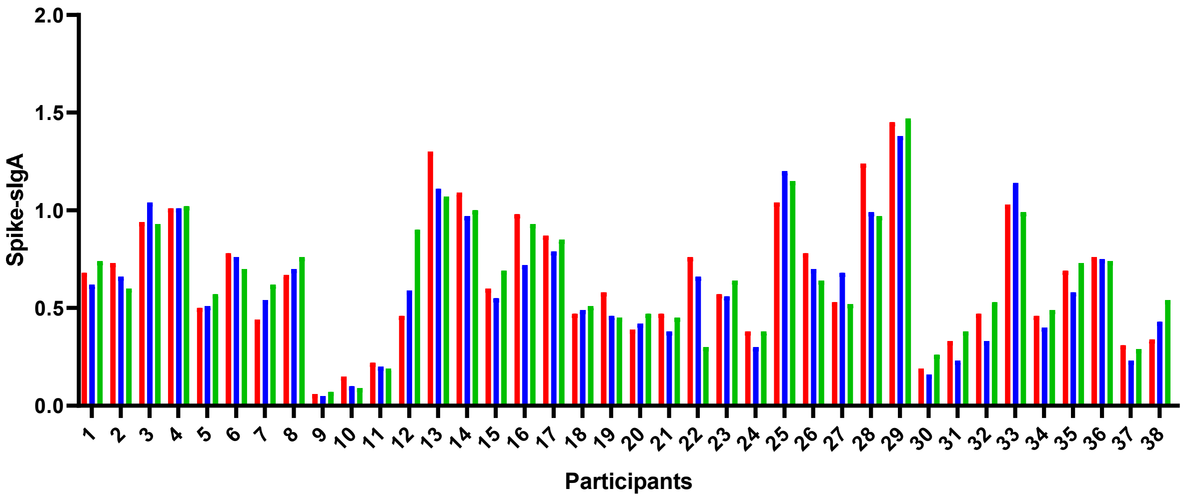

Figure S1. Spike IgA in human milk. Individual Spike IgA levels in UP milk (red), HPP milk (blue) and HoP milk (green).

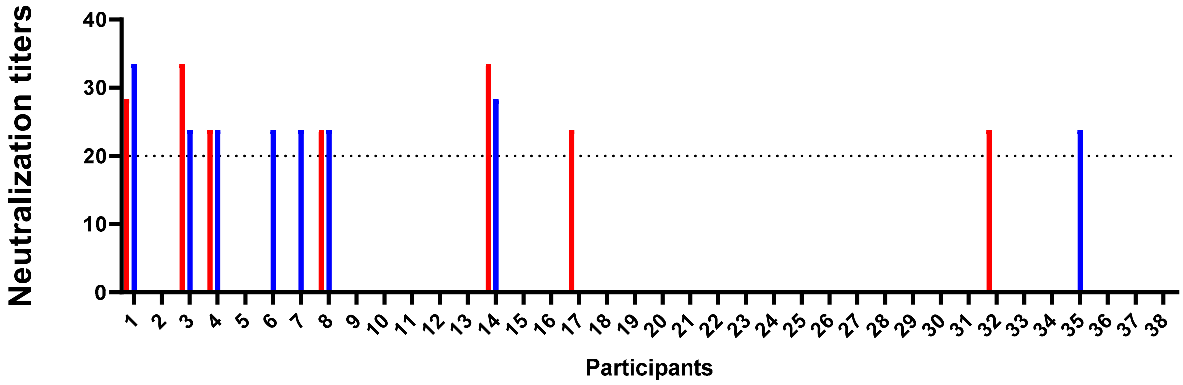

Figure S2. SARS-CoV-2 neutralizing capacity of human milk of a clinical isolate. Neutralization capacity in individual participants of UP milk (red), HPP milk (blue) and HoP milk (green).
